# Hydrothermal Synthesis Au-Bi_2_Te_3_ Nanocomposite Thermoelectric Film with a Hierarchical Sub-Micron Antireflection Quasi-Periodic Structure

**DOI:** 10.3390/ijms160612547

**Published:** 2015-06-03

**Authors:** Junlong Tian, Wang Zhang, Yuan Zhang, Ruiyang Xue, Yuhua Wang, Zhijian Zhang, Di Zhang

**Affiliations:** 1 State Key Laboratory of Metal Matrix Composites, Shanghai Jiao Tong University, Shanghai 200240, China; E-Mails: tianjunlong666@sjtu.edu.cn (J.T.); wlpnju@163.com (Y.Z.); xry1995@sjtu.edu.cn (R.X.); 2 Department of Prosthodontics, Shanghai Jiao Tong University, Shanghai 200240, China; E-Mail: heyaniy@hotmail.com; 3 Jushi Fiberglass Research Institute, Zhejiang Key Laboratory for Fiberglass Research, Jushi Group Co., Ltd., Hangzhou 314500, China; E-Mail: zjianzhang@163.com

**Keywords:** nanocomposite thermoelectric film, low-temperature hydrothermal synthesis, Au-Bi_2_Te_3_, antireflection quasi-periodic structure

## Abstract

In this work, Au-Bi_2_Te_3_ nanocomposite thermoelectric film with a hierarchical sub-micron antireflection quasi-periodic structure was synthesized via a low-temperature chemical route using *Troides helena* (Linnaeus) forewing (T_FW) as the biomimetic template. This method combines chemosynthesis with biomimetic techniques, without the requirement of expensive equipment and energy intensive processes. The microstructure and the morphology of the Au-Bi_2_Te_3_ nanocomposite thermoelectric film was analyzed by X-ray diffraction (XRD), field-emission scanning-electron microscopy (FESEM), and transmission electron microscopy (TEM). Coupled the plasmon resonances of the Au nanoparticles with the hierarchical sub-micron antireflection quasi-periodic structure, the Au-Bi_2_Te_3_ nanocomposite thermoelectric film possesses an effective infrared absorption and infrared photothermal conversion performance. Based on the finite difference time domain method and the Joule effect, the heat generation and the heat source density distribution of the Au-Bi_2_Te_3_ nanocomposite thermoelectric film were studied. The heterogeneity of heat source density distribution of the Au-Bi_2_Te_3_ nanocomposite thermoelectric film opens up a novel promising technique for generating thermoelectric power under illumination.

## 1. Introduction

Thermoelectric (TE) materials are of interest for potential use as heat dissipating devices for cooling hot-spots and generating electrical power from waste heat [1,2]. The efficiency of thermoelectric devices is determined by the TE materials’ dimensionless figure of merit, defined as:

(1) $$ZT=(S^{2}\text{σ}/k)T$$

where *S*, σ, *k* and *T* are the Seebeck coefficient, electrical conductivity, the thermal conductivity and absolute temperature, respectively [3]. Among numerous thermoelectric materials, bismuth telluride (Bi_2_Te_3_) and bismuth telluride-based alloys are the most important TE material, and exhibited the highest room-temperature figure of merit *ZT*~1 [4]. An excellent TE material should have a high σas a crystalline material and relatively low *k*, like glass, with the concept of the “phonon-glass, electron-crystal” model [2,5,6,7], where a disordered atomic arrangement is desirable for low lattice thermal conductivity (phonon-glass) but a crystalline semiconductor structure is desirable for the electronic properties (electron-crystal) [3]. Several possible approaches to enhancing *ZT* have been investigated. In addition to semiconductors with caged structures, for example skutterudites [8] and clathrates [9], the use of the fine microstructure, such as nano-inclusions in bulk matrices to form bulk nano-composite materials [1], the modification of the electron states with resonant impurities [10], or engineering band convergence [11], and strong electron–phonon coupling by charge density waves [12], nanostructures thermoelectric materials [7,13] is also regarded as “phonon-glass, electron-crystal” materials to enhance the efficiency of TE materials because of the reduction of *k* by the phonon-blocking effect of the nanostructures. The nanostructures approach has been demonstrated in the self-assembled quantum dot superlattices [13] and thin film [14] via nanostructuring in one- or two-dimensions [15].

Over the past few decades, a great number of TE film assembled by Bi_2_Te_3_ nanoparticles (NPs) were prepared by solvothermal synthesis combined with dip coating [14], electrodeposited [16,17,18], chemical vapor deposition [19], and so on. However, these techniques have severe restrictions on preparing a TE film assembled by NPs with a hierarchical sub-micron functional structure on macro-scale. In addition, these TE film just possess the single function of generating electrical power from heat or cooling from electric energy. Encouragingly, biomimetic gives a successful solution to overcome these restrictions [20,21,22,23,24]. Because nature provides us a multitude of remarkable biological materials, through billions of years of evolution, and biomimetics transfer optimum designs in nature to technical application. Among these a multitude of biological materials, the wings of butterflies attract more and more attention, mostly because the wings of butterflies possess typical sub-micron functional structures, such as multilayer [25], photonic crystal [26,27], ridge [28,29,30], irregular network [31], and window [32] that lead to advanced optical effects, including broad-angle structure color [33,34], color-mixing [25], polarization [25], iridescence [26], ultra-whiteness [31], antireflection and photoabsorption [28,29,30], and so on. Due to the advanced optical effects resulted from the complex and diverse structures, these butterfly wings were applied in the display of structural colors [33], surface-enhanced Raman scattering [35,36], advanced sensors [37,38], photochemical hydrogen production [39], infrared absorption [40], and solar cells [41].

Based on the antireflection and photoabsorption structure, the black butterfly wings exhibited extreme absorption in the visible range of the solar spectrum and an effective photothermal conversion performance, which uses the absorbed energy to warm up the muscle before flying [42,43]. Here, we combine the Au-Bi_2_Te_3_ nanocomposite thermoelectric film (ABTEF) with the antireflection and photoabsorption structure of black butterfly wings to prepare an Au-Bi_2_Te_3_ nanocomposite photo-thermal voltaic film (Au-Bi_2_Te_3__T_FW). In this work, ABTEF with a hierarchical sub-micron antireflection quasi-periodic structure (HSAQS) was synthesized via a low-temperature chemical route using *Troides helena* (Linnaeus) forewing (T_FW) as the biomimetic template. This method combines chemosynthesis with biomimetic techniques, without the requirement of expensive equipment and energy intensive processes. This combination presents new insight to take advantage of TE materials to generate electrical power from the solar thermal energy.

## 2. Results and Discussion

### 2.1. Morphology and Crystal Structure

**Figure 1:**
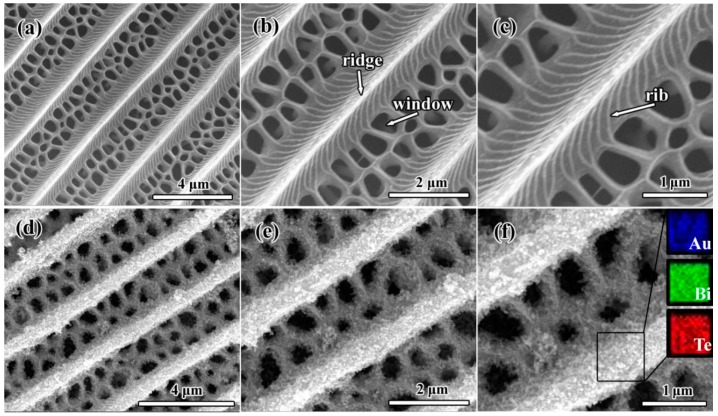
(**a**–**c**) SEM images of *Troides helena* (Linnaeus) forewing (T_FW); (**d**–**f**) SEM images of Au-Bi_2_Te_3__T_FW. The inset of (**f**) is the elemental maps showing the distribution of Au, Bi, and Te atoms on the surface of the hierarchical sub-micron antireflection quasi-periodic structure (HSAQS) of T_FW.

Scanning-electron microscopy (SEM) of the T_FW is shown in Figure 1a–c. As show in Figure 1a,b, along the length of the scale of the T_FW, it exhibited periodic triangular roof-type ridges and formed the periodic antireflection structure. The periodic triangular roof-type ridges are beneficial to focus light into the scale interior by multi-antireflection. We can also observe that staggered windows are present between every two ridges, which enhanced light-harvesting capacity. As shown in Figure 1c, declining microribs run down the sides of the clearly exhibited ridge. These microribs assist in the trapping of light due to the inducing of internal light scattering. The ridges, microribs and windows construct a hierarchical sub-micron antireflection quasi-periodic structure (HSAQS) that effectively traps light. The combination of a melanin/chitin composite with this HSAQS endows the black T_FW with an effective visible light trapping capability [28,40,42]. From the SEM images of Au-Bi_2_Te_3__T_FW (Figure 1d–f), these figures clearly exhibit that the Au-Bi_2_Te_3_ nanocomposite were deposited and agglomerated into thin film on the ridges and microribs. Moreover, the parallel periodic triangular roof-type ridges and windows of the T_FW were well maintained. The successful deposition of Au-Bi_2_Te_3_ nanocomposite onto the surface of the HSAQS of the T_FW was also confirmed by energy dispersive spectrometer (EDS) analysis, as shown in the inset of Figure 1f.

**Figure 2:**
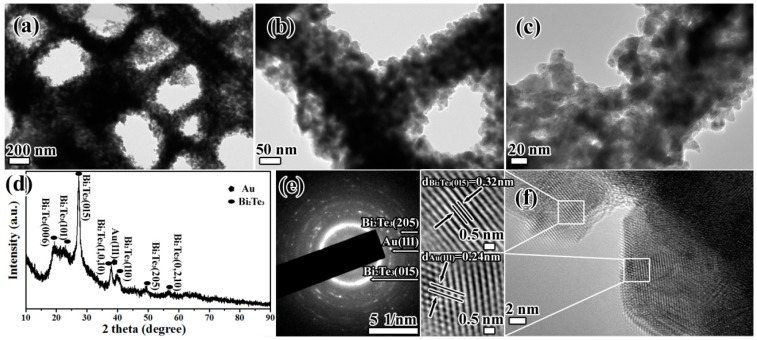
(**a**–**c**) TEM images of Au-Bi_2_Te_3__T_FW; (**d**) XRD result of Au-Bi_2_Te_3__T_FW; (**e**) SAED image of Au-Bi_2_Te_3__T_FW; (**f**) HRTEM image of Au-Bi_2_Te_3__T_FW.

Further insight was gained regarding the morphologies and microstructures of the Au-Bi_2_Te_3__T_FW, and the results are shown in Figure 2. From Figure 2a, we can find that the nanoparticles deposited on the surface of the HSAQS of T_FW. Moreover, the ridges and windows structures have been clearly maintained. As shown in Figure 2b,c, the Au-Bi_2_Te_3_ nanocomposites were deposited on the surface of the bio-template and agglomerated into a thin film. Furthermore, the deposition of Au-Bi_2_Te_3_ nanocomposites onto the T_FW was examined by X-ray diffraction (XRD). As revealed by the XRD results in Figure 2d, the diffraction peaks of Au-Bi_2_Te_3__T_FW are assigned to the (111) plane of cubic phase Au (JCPDS card No. 04-0784, Gold, syn), and (006), (101), (015), (1,0,10), (110), (205), and (0,2,10) planes of rhombohedral phase Bi_2_Te_3_ (JCPDS card No. 08-0027, Tellurobismuthite). Figure 2e is the selected area electron diffraction (SAED) image that displays ring and dot patterns corresponding to the major and minor phases in the product, respectively. The clear rings match well with the XRD results, and the relevant planes are indexed as (015) and (205) planes of the Bi_2_Te_3_, and (111) plane of Au, respectively. The lattice fringes with interplanar distance of *d*_Au(111)_ = 0.24 nm and *d*_Bi2Te3(015)_ = 0.32 nm are exhibited in the high resolution transmission electron microscope (HRTEM) image, which agrees with the results of the JCPDS card No. 04-0784 and JCPDS card No. 08-0027. These results further confirm the successful fabrication of the Au-Bi_2_Te_3__T_FW.

### 2.2. Optical Properties

**Figure 3:**
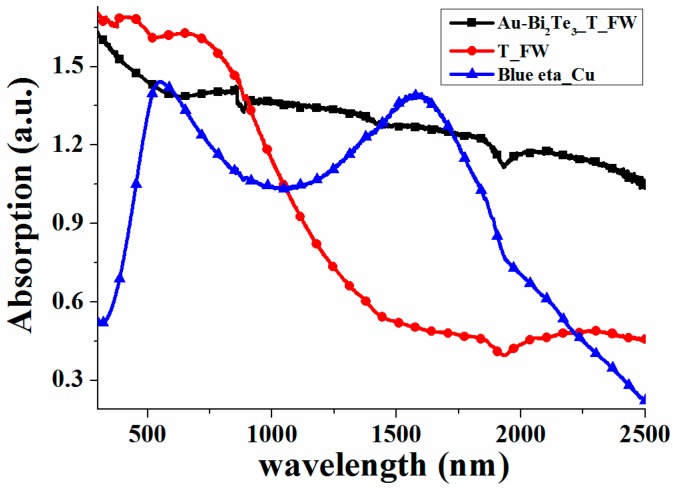
Absorption spectra of Au-Bi_2_Te_3__T_FW, T_FW and Blue eta_Cu over the wavelength range of 300–2500 nm.

The absorption spectra of Au-Bi_2_Te_3__T_FW, T_FW and BlueTec eta plus_Cu (Blue eta_Cu) over the wavelength range of 300–2500 nm is presented in Figure 3. Blue eta_Cu is a commercial absorber that functions as a solar thermal collector with an excellent solar absorption (0.945, AM 1.5) and effectively transforms solar energy into heat (BlueTec GmbH & Co KG, Hese, Germany) [44]. From Figure 3, we can find that the T_FW show excellent absorption over the visible and near infrared light region (300–909 nm) because of the coupling effect between the melanin/chitin composite and the HSAQS of the T_FW, which was demonstrated in our previous work [40]. Compared with the absorption of the T_FW, the Au-Bi_2_Te_3__T_FW possesses an enhanced infrared absorption due to plasmon resonance of the Au NPs and the coherent coupling between adjacent resonance systems integrated with the HSAQS of the T_FW [40]. Furthermore, compared with the Blue eta_Cu, Au-Bi_2_Te_3__T_FW exhibits a more effective light absorption performance over a wide spectral range, except in the wavelength ranges of 531–614 and 1443–1721 nm. The average absorbance intensities increased by 59.51% in the wavelength range of 300–2500 nm. These results demonstrate that the Au-Bi_2_Te_3__T_FW combined Au-Bi_2_Te_3_ nanocomposite with the HSAQS of the T_FW possesses an excellent light absorption performance over a wide spectral range, especially over the infrared range.

**Figure 4:**
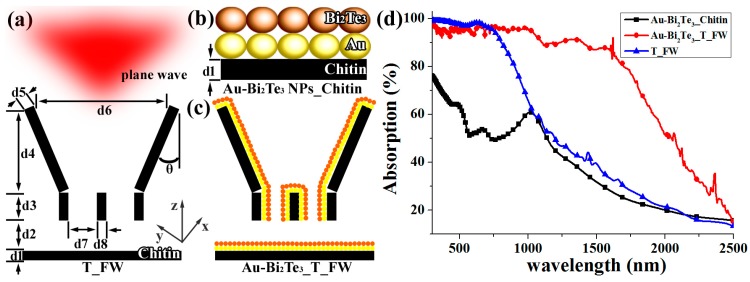
Models for FDTD simulation of (**a**) T_FW; (**b**) adding a layer of Au nanosphere array and a layer of Bi_2_Te_3_ nanosphere array to the surface of the chitin (Au-Bi_2_Te_3__Chitin) and (**c**) adding a layer of Au nanosphere array and a layer of Bi_2_Te_3_ nanosphere array to the surface of the HSAQS of the T_FW (Au-Bi_2_Te_3__T_FW); (**d**) FDTD calculations for the absorption spectra of Au-Bi_2_Te_3__T_FW, Au-Bi_2_Te_3__Chitin and T_FW.

To further demonstrate that the Au-Bi_2_Te_3__T_FW combined Au-Bi_2_Te_3_ nanocomposite with the HSAQS of the T_FW possesses an excellent light absorption performance over a wide spectral range, the finite difference time domain method (FDTD) simulation was used. Models for FDTD simulation of T_FW and dimensions of d1–d8 and θ were offered in our previous work, which are based on careful observation and statistical analysis of several SEM and TEM images of T_FW [40]. The diameter of the Au nanosphere and Bi_2_Te_3_ nanosphere are set to 20 nm. In order to use FDTD to simulate the Au-Bi_2_Te_3__Chitin, a layer of Au nanosphere array and a layer of Bi_2_Te_3_ nanosphere array were added to the surface of the chitin structured the FDTD simulation model. The FDTD simulation model of Au-Bi_2_Te_3__T_FW was constructed by adding a layer of Au nanosphere array and a layer of Bi_2_Te_3_ nanosphere array to the surface of the HSAQS of the T_FW. From the FDTD calculations (Figure 4d), we can find that Au-Bi_2_Te_3__T_FW exhibits an enhancement in absorption over a broadband infrared region, compared with the absorption spectra of T_FW. However, compared with the absorption spectra of T_FW, the Au-Bi_2_Te_3__T_FW exhibits a lower absorption over the visible and near infrared light region, as the melanin/chitin composite possessed an excellent absorption performance over the visible light region [29,42]. These findings indicate that the Au-Bi_2_Te_3_ nanocomposite array can enhance infrared absorption over a broadband. Additionally, compared with the absorption spectra of Au-Bi_2_Te_3__Chitin, that of Au-Bi_2_Te_3__T_FW exhibits a more intensive absorption over the wavelength range over 300–2500 nm. These results demonstrate that Au-Bi_2_Te_3_ nanocomposite, integrated with the HSAQS, can achieve an enhanced broadband light absorption. These results of FDTD calculations are in excellent agreement with those observed experimentally, as shown in Figure 3 and Figure 4d.

### 2.3. Photothermal Conversion Properties

**Figure 5:**
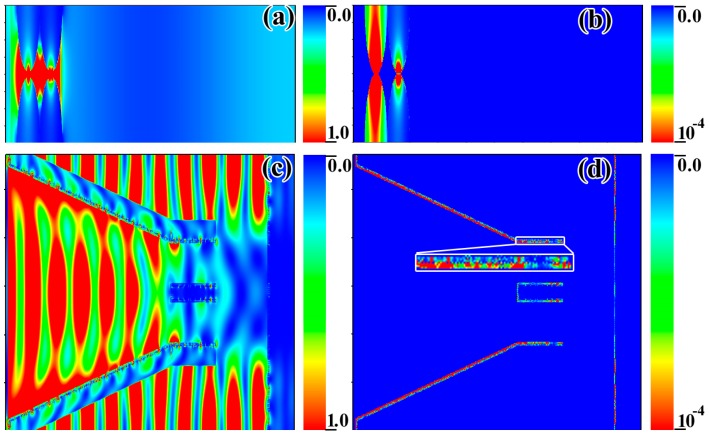
(**a**,**c**) ${\left|\vec{E}\right|}^{2}$ intensity distribution maps of Au-Bi_2_Te_3__Chitin and Au-Bi_2_Te_3__T_FW, respectively; (**b**,**d**) heat source density maps of Au-Bi_2_Te_3__Chitin and Au-Bi_2_Te_3__T_FW, respectively. The wavelength of the incident light is fixed under 980 nm.

Based on the FDTD method and the Joule effect [45], we discuss the heat generation of Au-Bi2Te3_Chitin and Au-Bi2Te3_T_FW under illumination. Based on the FDTD method, the ${\left|\vec{E}\right|}^{2}$ intensity distribution has been simulated. Moreover, the ${\left|\vec{E}\right|}^{2}$ intensity distribution maps of Au-Bi_2_Te_3__Chitin and Au-Bi_2_Te_3__T_FW are shown in Figure 5a,c, respectively, in which the wavelength of the incident light is fixed under 980 nm. From Figure 5a, we can find that a more intensive electric field intensity is located in the adjacent regions between two nanospheres, providing electric field hotspots, especially in the interparticle region. This finding demonstrates that the adjacent interaction of the Au-Bi_2_Te_3_ nanocomposites can substantially enhance the electric field in the adjacent region. As shown in Figure 5c, the intensive electric field distributed on the surface of the Au-Bi_2_Te_3_ nanocomposites, which covered the surface of the ridges of the HSAQS, and is distributed in between two ridges of the HSAQS. These findings demonstrate that the periodic triangular roof-type ridges form the periodic antireflection structure, which focuses light into the scale interior, and that the HSAQS can trap light effectively [40]. In addition, the Au-Bi_2_Te_3_ nanocomposites and the adjacent interaction of the Au-Bi_2_Te_3_ nanocomposites integrated with the HSAQS can further enhance the light absorption. When a plasmonic structure is under illumination, the heat source density arises from the Joule effect, and that the heat source density can be expressed as a function of the electric field [45]:

(2) h(r→)=ωε0Im(εω)|E(r→)|2

where ω is the angular frequency of the light, $\varepsilon_{\omega }$ is the permittivity of the material, and $E(\vec{r})$ is the electric field. Based on the ${\left|\vec{E}\right|}^{2}$ intensity distribution (Figure 5a,c) obtained by FDTD simulation, we study the heat source density distribution of the Au-Bi_2_Te_3__Chitin and Au-Bi_2_Te_3__T_FW, respectively (Figure 5b,d). As shown in Figure 5b, the heat arises mainly from the photothermal material (Au NPs and Bi_2_Te_3_ NPs). In addition, more intensive heat source density distributes on the adjacent region between two plasmonic structures. This finding demonstrates that the coherent coupling between adjacent resonant systems enhance hot power yield. As shown in Figure 5d, the heat arises from the photothermal material (Au NPs and Bi_2_Te_3_ NPs), which covers the surface of the HSAQS of the T_FW. Additionally, we can find that the most of hot power yields of the Au-Bi_2_Te_3__T_FW arise from the photothermal materials covering the surface of the ridges of the T_FW. Because the heat source density on the surface of the ridges of the T_FW are more intensive compared with the intensity of heat source density on the surface of the windows of the T_FW. In addition, the intensity of the heat source density on the surface of the windows of the T_FW decreased with the increased the depth of the window, as shown in the inset of Figure 5d. These findings demonstrate that the heat source density distribution of the Au-Bi_2_Te_3__T_FW under illumination, is clearly nonuniform. Under illumination, the nonuniformity of the heat source density distribution of the TE film (Au-Bi_2_Te_3__T_FW) will be beneficial to generate electrical power. Consequently, the Au-Bi_2_Te_3__T_FW can potentially be used to generate electrical power from the solar thermal energy or micro region themoelectric energy production illuminated by infrared, due to the nonuniformity of the heat source density distribution of the TE film.

## 3. Experimental Section

### 3.1. Materials

*T. helena* butterflies were obtained from Shanghai Natural Wild-Insect Kingdom Co., Ltd. (Shanghai, China). Absolute ethanol (EA, 97%) was purchased from Changshu Yangyuan Chemical Co., Ltd. (Changshu, China). Nitric acid (NA, 67%), ethylenediamine (ED, 99%), Tellurium dioxide (TeO_2_), Potassium hydroxide (KOH), Bisumuth trichloride (BiCl_3_), and Sodium tetrahydroborate (NaBH_4_) were purchased from Sinopharm Chemical Reagent Co., Ltd. (Shanghai, China). All of these compounds are analytically pure and were used as received without further purification.

### 3.2. Synthesis of Au-Bi_2_Te_3__T_FW

**Figure 6:**
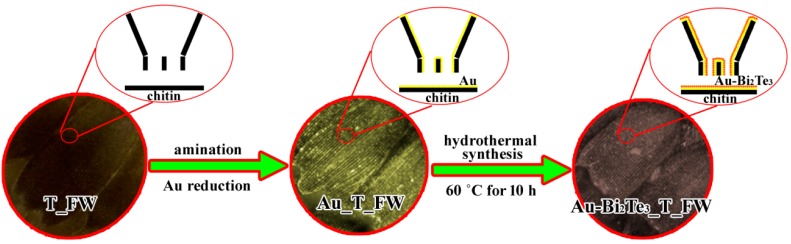
Schematic representation of the procedure of fabricating of Au-Bi_2_Te_3__T_FW.

The procedure of fabricating the Au-Bi_2_Te_3__T_FW includes two steps (Figure 6). Firstly, to prepare the Au butterfly wing (Au _T_FW), T_FW were first immersed in diluted 8 vol % nitric acid for 2 h, and then washed in deionized water. The wings were then immersed in an ethanol solution of ethanediamine (25 vol %) for 6 h to obtain the aminated T_FW, and then washed with ethanol and deionized water. In succession, the aminated T_FW was immersed in an aqueous solution of HAuCl_4_ (0.2 wt %) for 10 h, washed with deionized water, then dipped in an aqueous solution of NaBH_4_ (0.1 mol/L) for 15 min, and rinsed in deionized water [35]. Upon completion of these steps, Au_ T_FW was fabricated. The above dipping process was done in a 30 °C constant temperature water bath. Secondly, the Au_T_FWs was put into the mixture, impregnant and sealed in an autoclave, and heated at 60 °C for 10 h. After being cooled down to room temperature, the treated wings were taken out and washed with deionized water and ethanol. Finally, the as-obtained wings were dried thoroughly *in vacuum* at 25 °C to obtain the target sample Au-Bi_2_Te_3__T_FW. The impregnated mixture is composed of BiCl_3_·2H_2_O (10 mmol), TeO_2_ (15 mmol), KOH (80 mmol), NaBH_4_ (30 mmol), and deionized water (70 mL).

### 3.3. Finite Difference Time Domain Method (FDTD) Simulation

The detailed FDTD model of T_FW, with an added layer of Au nanosphere array and a layer of Bi_2_Te_3_ nanosphere array to the surface of the chitin (Au-Bi_2_Te_3__Chitin), and an added layer of Au nanosphere array and a layer of Bi_2_Te_3_ nanosphere array to the surface of the HSAQS of the T_FW (Au-Bi_2_Te_3__T_FW), are shown in Figure 4. All simulations were performed under normal incident light, with a plane wave light source laid above the ridges at a distance of 500 nm, and the red arrow represents the incidence direction of the light. The reflection (R) monitor is located behind the light source at a distance of 500 nm. The transmission (T) monitor is located 100 nm beneath the model (Figure 2d). The absorption is deduced from A = 1 − T − R. The boundary condition in the *y* direction is periodic (periodic boundary condition, PBC), and in the *x* direction is absorbing (perfectly matched layer, PML). The span of *z* direction is zero [40]. In our simulation, the complex permittivity index of the Au, chitin, and Bi_2_Te_3_ are provided by the material database of the Optiwave and the reported results [46,47], respectively. The mesh size was chosen to obtain a good tradeoff between the computer memory required and the simulation time, while ensuring convergence of the results. A convergence test was carefully performed.

### 3.4. Characterization

Characterization using a scanning electron microscope (SEM) was performed on a 20-kV field emission SEM (FESEM) instrument (Quanta 250, FEI, Hillsboro, OR, USA). X-ray diffraction measurements were conducted using a Rigaku D/max-2550 instrument, equipped with a Cu-Kα radiation source (Rigaku Corp., Tokyo, Japan). Transmission electron microscope (TEM), high resolution transmission electron microscope (HRTEM), and selected area electron diffraction (SAED) measurements were performed on a JEM-2100F transmission electron microscope (JEOL, Peabody, MA, USA) operated an acceleration voltage of 200 kV. The absorption over the wavelength range of 300–2500 nm was measured using a Lambda 750 UV–VIS–NIR spectrophotometer (PerkinElmer, Waltham, MA, USA). For UV–VIS–NIR spectroscopy, the samples were mounted directly using the clip, which is located behind the integrating sphere without any substrates.

## 4. Conclusions

In this work, we explored a straightforward and low-cost method for fabricating ABTEF with a HSAQS on a macroscopic centimeter-scale via a low-temperature chemical route using T_FW as the biomimetic template. The Au-Bi_2_Te_3__T_FW was combined with an Au-Bi_2_Te_3_ nanocomposite and the adjacent interaction of the Au-Bi_2_Te_3_ nanocomposites with the HSAQS of the T_FW, and achieved a drastically enhanced absorption over a broad spectral range, especially, over the NIR region. Under illumination, the nonuniformity of the heat source density distribution of the TE film (Au-Bi_2_Te_3__T_FW) will be beneficial to generate electrical power. Consequently, the Au-Bi_2_Te_3__T_FW can potentially be used to generate electrical power from solar thermal energy or micro region themoelectric energy production illuminated by infrared, due to the nonuniformity of the heat source density distribution of the TE film. This work presents new insight in taking advantage of TE materials to generate electrical power from the solar thermal energy. Moreover, this work, therefore, provides a versatile tool for fabricating photothermoelectric film, which is combined the submicron photonic architecture with nanocomposite (plasmon-TE material).
